# Effects of heliox as carrier gas on ventilation and oxygenation in an animal model of piston-type HFOV: a crossover experimental study

**DOI:** 10.1186/1475-925X-9-71

**Published:** 2010-11-12

**Authors:** Bakhtiyar Zeynalov, Takehiko Hiroma, Tomohiko Nakamura

**Affiliations:** 1 Division of Neonatology, Nagano Children's Hospital, Azumino City, Nagano, Japan; 2 Department of Pediatrics, Tokyo Medical & Dental University, Tokyo, Japan

## Abstract

**Objective:**

This study aimed to compare gas exchange with heliox and oxygen-enriched air during piston-type high-frequency oscillatory ventilation (HFOV). We hypothesized that helium gas would improve both carbon dioxide elimination and arterial oxygenation during piston-type HFOV.

**Method:**

Five rabbits were prepared and ventilated by piston-type HFOV with carrier 50% helium/oxygen (heliox50) or 50% oxygen/nitrogen (nitrogen50) gas mixture in a crossover study. Changing the gas mixture from nitrogen50 to heliox50 and back was performed five times per animal with constant ventilation parameters. Arterial blood gas, vital function and respiratory test indices were recorded.

**Results:**

Compared with nitrogen50, heliox50 did not change PaCO_2 _when stroke volume remained constant, but significantly reduced PaCO_2 _after alignment of amplitude pressure. No significant changes in PaO_2 _were seen despite significant decreases in mean airway pressure with heliox50 compared with nitrogen50.

**Conclusion:**

This study demonstrated that heliox enhances CO_2 _elimination and maintains oxygenation at the same amplitude but with lower airway pressure compared to air/O_2 _mix gas during piston-type HFOV.

## Background

Helium is a noble gas with very low atomic weight (4 g/mol) and density (0.18 g/L). When mixed with oxygen as heliox, the low density of helium reduces the resistance associated with gas delivery. This increased mobility has three effects: gas more readily reaches the alveoli, allowing greater diffusion; breathing effort is significantly reduced with use of a less-dense gas; and carbon dioxide (CO_2_) is eliminated more rapidly [1-5].

High-frequency oscillatory ventilation (HFOV) offers a tempting advantage in maintaining oxygenation using higher mean airway pressure with minimal risk of complications. HFOV has been used in a variety of clinical situations, including neonatal respiratory distress syndrome, congenital diaphragmatic hernia, meconium aspiration syndrome, and air leak syndrome. Heliox has been reported as beneficial with various forms of nonconventional ventilation, including HFOV, high-frequency jet ventilation (HFJV), and high-frequency percussive ventilation. Winters et al. described a case series of 5 pediatric patients who showed marked improvements in ventilation with the use of heliox during HFOV [6]. Gupta et al. reported combined use of heliox and HFJV to enhance CO_2 _elimination in a 5-month-old infant with acute respiratory failure, gas-trapping, hypercarbia, respiratory acidosis, and air leak syndrome [7]. Stucki et al. reported successful use of heliox with high-frequency percussive ventilation in a 5-year-old boy with cystic fibrosis and severe acute respiratory failure [5]. Katz et al. used an in vivo model of acute lung injury and found that high-frequency membrane-type oscillator ventilation with heliox improved oxygenation and CO_2 _elimination. A subsequent study by the same authors using the same animal model and ventilator showed that the improvements seen in the first study were related to larger tidal volume (Vt) delivery by the oscillator with heliox [3,4]. However, significant differences appear to exist between the effects of different types of oscillator [8-11]. The effects of heliox on gas exchange and oxygenation during piston-driven HFOV in animals or humans have not been studied sufficiently in a prospective manner. The goal of our study was to examine the effects of heliox on gas exchange and oxygenation in an animal model during high-frequency piston-type oscillatory ventilation. We hypothesized that, compared with oxygen-enriched air, heliox would improve both CO_2 _elimination and arterial oxygenation.

## Method

All study protocols were approved by the Institutional Animal Care and Use Committee of Nagano Children's Hospital, Nagano, Japan.

Five Japanese white rabbits (body weight, 1.96 ± 0.02 kg) were used in our study. Animals were premedicated by intramuscular administration of ketamine hydrochloride (10 mg/kg/dose) and xylazine (5 mg/kg/dose). The ear vein was cannulated using a 24-G peripheral angiocatheter for intravenous anesthesia and hydration. Animals were placed in a supine position under a radiant warmer to maintain body temperature during the entire study period, and body temperature was monitored using a rectal temperature probe. Tracheotomy was performed, and a no-cuff endotracheal tube (ETT) with an internal diameter of 3.0 mm (Portex, London, UK) was inserted to a depth of 4 cm and fixed in place. Intermittent ventilation (IMV) was initiated using a time-cycled, pressure-limited commercial ventilator (Humming II; Metran, Saitama, Japan) with: FiO_2_, 0.5; inspiration time (Ti), 0.6 s; positive end expiratory pressure, 5 cmH_2_O; peak inspiratory pressure, 14 ± 1 cmH_2_O, sufficient to achieve a Vt of 10 ml/kg; and respiratory rate, 20 breaths/min. Vt was measured during the experiment proper using a low dead space, hot-wire pneumotachograph (LFM-317 Aivision Laminar Flow Meter; Metabo, Lausanne, Switzerland). The carotid artery was then cannulated for direct blood pressure measurement, heart rate (HR) monitoring and determination of arterial blood gases (ABG). Anesthesia and myoparalysis were provided by continuous intravenous infusion of ketamine hydrochloride (5 mg/kg/h) and pancuronium (0.1 mg/kg/h). Arterial oxygen saturation was monitored continuously using a transcutaneous pulse oximeter (DDG2001; Nihon Kohden, Tokyo, Japan). These data were displayed on a monitor throughout the experiments and recorded to a computer for subsequent analysis. ABG were analyzed (I-STAT portable clinical analyzer; I-STAT, East Windsor, NJ, USA) under the protocol, and then values were corrected to body temperature. Animal hydration was maintained by infusion of 0.9% sterile NaCl from the beginning of anesthesia at 20 ml/kg over 30 min, and at 3 ml/kg/h throughout the whole experiment. Animals were allowed 20 min for stabilization, and then baseline recordings were taken and the animal was shifted to the HFOV mode of the same ventilator (Humming II; Metran). ABG were obtained after 10 min of ventilation with stroke volume (SV) 16 mL, which is respirator setting determining airway amplitude pressure (AMP), mean airway pressure (MAP) 15 cmH_2_O, frequency 15 Hz and FiO_2 _0.5. Each animal was then switched to heliox50 and ventilated for 10 min before obtaining data. After data collection with heliox50, the animal was returned to nitrogen50 and data were collected. The animal was subsequently changed to heliox50 and allowed 10 min to stabilize before data collection. Ventilator settings were held constant while the gas mixture was changed. The cycle of changing the gas mixture from nitrogen50 to heliox50 and back, with constant parameters of ventilation, was performed three times per animal. Using a digital pressure sensor (AP-C40, KEYENCE, Osaka, Japan) installed into the Y-piece of the breathing circuit, actual pressure parameters were measured and registered. Animals were switched to heliox50 during the fourth cycle, and ventilated to adopt the same AMP just before nitrogen50. ABG and monitor data were also recorded. In the fifth cycle, to ensure that AMP returned to baseline amplitude after removal of heliox, rabbits were ventilated at SV 16 ml with nitrogen50. Finally, ABG and vital function indices were recorded.

### Statistical Analysis

Experimental data have been accumulated in a spreadsheet application (Excel 2003; Microsoft, WA, USA). All results are expressed as means ± standard deviation. Experimental data were analyzed using nonparametric statistical methods and the Wilcoxon signed-ranks test. Values of p ≤ 0.05 were considered statistically significant.

## Results

MAP, AMP, PaO_2 _and PaCO_2 _values during the experiment are summarized in Table 1.

**Table 1:** MAP, AMP, PaO_2 _and PaCO_2 _values during the experiment, FiO_2_, 0.5; Frequency, 15 Hz, Values represent mean ± SD. * P < 0.05,

Experimental series	1	2	3	4	5
**Carried Gas**	**Nitrogen50**	**Heliox50**	**Nitrogen50**	**Heliox50**	**Nitrogen50**
**SV (mL)**	**16**	**16**	**16**	**25**	**16**

MAP (cmH_2_O)	14 ± 0.55	11.5 ± 0.72*	13.8 ± 0.6	11.9 ± 0.85*	13.8 ± 0.88
AMP (cmH_2_O)	40.1 ± 0.61	28.7 ± 0.51*	39.8 ± 1.22	38.4 ± 0.95	39.8 ± 0.61
PaO_2 _(mmHg)	207 ± 8.6	196 ± 15.5	210 ± 26.8	204 ± 18.4	214 ± 24.3
PaCO_2 _(mmHg)	43 ± 13	47 ± 14	45 ± 11	33 ± 5*	43 ± 6

With nitrogen50 ventilation, with a setting of SV 16 ml and frequency 15 Hz, monitoring values were MAP 14 ± 0.55, 13.8 ± 0.6 and 13.8 ± 0.88 cmH_2_O, and AMP 40.1 ± 0.61, 39.8 ± 1.22 and 39.8 ± 0.61 cmH_2_O, respectively. The shift from nitrogen50 to heliox50 was accompanied by significant low in MAP and AMP to maintain the same PaO_2 _and PaCO_2_. Heliox50 thus significantly reduced PaCO_2 _after alignment of AMP.

## Discussion

Heliox has been examined previously in healthy [2,12] and injured animals [3,4] using membrane-type high-frequency ventilation, resulting in minimal improvements in CO_2 _elimination. Our crossover investigation of the use of heliox during HFOV in a rabbit model with healthy lungs revealed significant decreases in MAP and AMP to maintain stable oxygenation and PaCO_2_.

Several papers have described significant differences between using different types of high-frequency ventilators [8-11]. Those studies examined not only different types of high-frequency ventilation, but also different types of oscillators. Differences were identified between oscillatory pressure waveforms and the efficiency of volume delivery, both between and within each ventilator category. Jouvet et al. observed differences in the relationship between Vt and AMP, and found that the membrane-type ventilator had a high volume output, and may deliver "supraphysiological" Vt [11]. Pillow et al. [9] also used an in vitro model to show that HFO performance differs according to ventilator properties. Ventilator-displayed amplitude and airway-opening amplitude are closely related in piston- and membrane-type ventilators, but in the case of membrane-type HFOV, airway-opening amplitude was typically 50% greater than ventilator-displayed amplitude. Vt has been shown to decrease in both types of ventilator when frequency increased under constant ventilator-displayed amplitude. However, comparing Vt between piston- and membrane-type ventilators showed that at the same frequency, Vt was significantly lower for piston-type ventilators. Conversely, Hatcher et al. [8] reported that during piston-type HFOV, CO_2 _transport increased in a linear manner with increasing frequency, while a membrane-type oscillator showed decreasing CO_2 _transport with increasing frequency. They speculated that piston-type oscillators are able to maintain stroke volume over a range of frequencies. Mildner et al. [10] evaluated current membrane- and piston-type devices using in vitro modeling with heliox and room air as carrier gases. They showed that speed of CO_2 _transport increased in a linear manner with increasing frequency when using air as the carrier gas for both types of ventilator. Use of heliox was followed by significant increases in CO_2 _transport, but increases depended on frequency and the device. Augmentation of CO_2 _transport with heliox was greatest at 5 Hz for the piston-type device, and 15 Hz for the membrane-type ventilator [10]. Heliox may alter gas exchange during HFOV through a number of mechanisms. As helium is less dense than nitrogen, the frictional forces in turbulent flows are reduced with heliox as compared with oxygen-enriched air. For a given set of airway dimensions, turbulent flow results in a higher resistance than laminar flow. In addition, mechanical ventilation through a narrow ETT and airway, particularly in pediatric and neonatal patients, may further increase the Reynolds number and thus increase turbulent flow [13,14]. Therefore, with heliox, the calculated Reynolds number is lower, which may change regions of turbulent flow to laminar flow, reducing resistance and energy loss. As resistive forces and energy waste are decreased, Vt per oscillation increases. We think that the effect of increasing Vt is dominant. This theory has been confirmed before in a laboratory study [2]. With Vt maintained at a constant level, no improvement in gas exchange was detected when combining heliox with HFOV. The precise mechanisms of gas exchange during HFOV are complex and seem to be largely unelucidated [15]. In theory, because of the lower density, heliox may favorably alter gas exchange through the pendelluft effect, inhalation/exhalation flow asymmetry, Taylor dispersion, and molecular diffusion.

## Conclusion

This study demonstrated that heliox enhances CO_2 _elimination and maintains oxygenation with the same amplitude but lower airway pressure compared to air/O_2 _mix gas during piston-type HFOV.

## Competing interests

The authors declare that they have no competing interests.

## Contributions

ZF; participated in the design of the study and performed the statistical analysis.

TH; participated in the design of the study and performed the statistical analysis.

TN; conceived of the study, and participated in its design and coordination and helped to draft the manuscript.

All authors read and approved the final manuscript.
